# Paracetamol in Patent Ductus Arteriosus Treatment: Efficacious and Safe?

**DOI:** 10.1155/2017/1438038

**Published:** 2017-07-30

**Authors:** Flaminia Bardanzellu, Paola Neroni, Angelica Dessì, Vassilios Fanos

**Affiliations:** Neonatal Intensive Care Unit, Neonatal Pathology and Neonatal Section, AOU and University of Cagliari, Cagliari, Italy

## Abstract

In preterm infants, failure or delay in spontaneous closure of Ductus Arteriosus (DA), resulting in the condition of Patent Ductus Arteriosus (PDA), represents a significant issue. A prolonged situation of PDA can be associated with several short- and long-term complications. Despite years of researches and clinical experience on PDA management, unresolved questions about the treatment and heterogeneity of clinical practices in different centers still remain, in particular regarding timing and modality of intervention. Nowadays, the most reasonable strategy seems to be reserving the treatment only to hemodynamically significant PDA. The first-line therapy is medical, and ibuprofen, related to several side effects especially in terms of nephrotoxicity, is the drug of choice. Administration of oral or intravenous paracetamol (acetaminophen) recently gained attention, appearing effective as traditional nonsteroidal anti-inflammatory drugs (NSAIDs) in PDA closure, with lower toxicity. The results of the studies analyzed in this review mostly support paracetamol efficacy in ductal closure, with inconstant low and transient elevation of liver enzymes as reported side effect. However, more studies are needed to confirm if this therapy shows a real safety profile and to evaluate its long-term outcomes, before considering paracetamol as first-choice drug in PDA treatment.

## 1. Introduction

Ductus Arteriosus (DA) is the vascular communication connecting pulmonary artery to aorta and represents one of the fundamental shunts of prenatal life circulation [1, 2].

The expression “Patent DA” describes the situation of a physiological or pathological open DA, which we will consider in this review with the abbreviation “PDA.” The conditions of “Patent DA” and “Persistent DA” are often confused and indicated with the same general expression “PDA,” even if they differ in terms of morphology, clinical effects, and management [1, 3, 4]. In healthy full-term newborns DA generally undergoes functional closure between 24 and 72 hours of life [5, 6], favored by higher postnatal levels of PaO2, removal of placenta from neonatal circulation, increase in pulmonary flow, and decline of prostaglandin E2 (PGE2) local receptors number [7, 8], reducing PGE2 vasodilating effect on DA [9–11].

The effect of PGE2 on vascular smooth muscle relaxation results in maintenance of ductal patency and occurs through activation of adenylate cyclase, leading to cAMP increase, via interaction with G-protein receptors [12, 13].

The anatomical closure of DA is generally complete in a few weeks [9], through the evolution into the structure called ligamentum arteriosum [14].

For several reasons, including the persistence of high levels of circulating PGE2 in preterm neonates, spontaneous DA closure often fails or is delayed in such patients, and this condition has been associated with various and severe short- and long-term complications [2, 6].

According to Benitz and Committee on Fetus and Newborn [6], on the 4th day of life PDA would persist in about 10% of infants with gestational age (GA) between 30 and 37 weeks, in 80% of those with GA between 25 and 28 weeks and in 90% of preterms born at 24 weeks of GA. From the 7th day of postnatal life the percentage of infants with PDA in these groups would reduce, respectively, to about 2%, 65%, and 87%.

According to the same review, DA would spontaneously close in 73% of infants with more than 28 weeks of GA and in 94% of infants with birth weight (BW) greater than 1000 grams [6].

There are few studies on PDA spontaneous closure in newborns with lower GA and BW, or in infants with respiratory distress syndrome (RDS), because a PDA therapeutic closure is often performed in these categories of patients. In a randomized trial comparing prophylactic indomethacin to placebo, non-pharmacologically treated PDA has not led to the development of clinical effects in 50% of infants of BW between 500 and 999 grams [6].

The spontaneous closure of PDA during early postnatal life in 35% of ELBW infants and in 70% of infants with GA greater than 28 weeks has been demonstrated in a prospective study by Koch et al. [15]; in another study, 75% of infants with GA less than 27 weeks with PDA at discharge moment has shown spontaneous ductal closure within the first year of life [5, 16].

Prolonged condition of PDA in preterms can be associated with important complications, such as severe RDS, prolonged need for assisted ventilation, pulmonary hemorrhage, bronchopulmonary dysplasia (BDP) [17], necrotizing enterocolitis (NEC), renal function damage, intraventricular hemorrhage (IVH), periventricular leukomalacia (PLV), cerebral palsy, or death [1, 14, 18–22].

These conditions depend on the magnitude of left-right shunt volume through PDA, regulated by the balance between PDA dimension and arterial resistance fall in the pulmonary circle during the early hours of postnatal life and resulting in lung hyperflow and development of pulmonary congestion and edema. If this condition persists, deterioration of respiratory function can occur. The impact of this “ductal steal” on systemic circulation causes a reduction in cardiac output increasing, the mechanism that allows facing the rising in systemic resistances of postnatal period. This condition can lead to vital organs perfusion impairment, such as brain, kidney, and bowel [6, 9].

To prevent such complications, the practice of DA closure is common and it is performed at first pharmacologically, but, in case of drugs failure or contraindication, with surgical ligation [18].

Despite years of researches and clinical experience on PDA management, many unresolved issues about its evaluation and treatment, with consequent heterogeneity of clinical practices in different centers, still remain, particularly regarding timing and modality of intervention. In fact, the available strategies vary from prophylactic treatment to early or delayed therapy [6, 23].

Recent studies, however, do not recommend prophylaxis in case of non-hemodynamically significant PDA, because it exposes the infants to indomethacin or ibuprofen adverse effects, without substantial short-term or long-term benefits [1, 12, 14]. The most reasonable strategy seems to be, nowadays, reserving the treatment only to hemodynamically significant PDA (hsPDA) [5, 24].

For this purpose, the first-line therapy is medical and nonsteroidal anti-inflammatory drugs (NSAIDs) are drugs of choice, preventing the conversion of arachidonic acid into prostaglandins via cyclooxygenase (COX) inhibition, in both the existing isoforms COX-1 (constitutive) and COX-2 (inducible) [18, 23, 25]. Reduction in prostaglandin levels leads to DA muscular wall constriction through the hypoxia of ductal vasa vasorum and consequent local angiogenesis, formation of neointimal tissue, and apoptosis. These mechanisms, in conjunction with platelet recruitment and activation, lead to processes of obstruction and fibrosis and, as a result, anatomical ductal closure [26–30].

## 2. Biochemical Markers of PDA

Many biochemical markers have been correlated with PDA such as B-type Natriuretic Peptide (BNP), the segment of the amino terminal B-type Natriuretic Peptide (NT-proBNP), and the cardiac Troponin T (cTnT), whose levels increase in case of hsPDA with right to left shunt, and could help in disease staging and management [5, 12]. Rising levels of BNP could represent a compensatory diuretic mechanism facing the increase in cardiac preload induced by the hyperaldosteronism condition subsequent to renal hypoperfusion and activation of renin-angiotensin system.

El Kuffash et al. [31] and Czernik et al. [32] also evaluated urinary proBNP as a simple and noninvasive PDA indicator, becoming higher in ventilated neonates nonresponders to treatment; according to Vettukattil [33] urinary NT-proBNP-to-creatinine ratio may be related to medical treatment response.

However, not all the authors agree with these results. Rostas and McPherson [34] affirm that BNP and NT-proBNP are not effective and really useful biomarkers to orient PDA therapy.

Some studies have also pointed out a role of systemic inflammation, which could improve COX-1 activity and PGE2 production, in ductal patency maintenance [35, 36]; this inflammatory pathway could also play a negative role influencing drug therapy response [37, 38].

In this perspective, Hillman et al. [35] performed the first study (on a sample of 132 newborns), investigating high sensitivity C-Reactive Protein (CRP) levels, as marker of low grade inflammation [39], detecting significantly higher levels of this mediator in patients diagnosed for PDA [35]; successively, Meinarde et al. [40] detected the same result analyzing 88 newborns.

These findings, though detected on small samples of patients, focus on the possible influence of inflammatory conditions on PDA, also in case of occurrence in prenatal life (such as chorioamnionitis) [35, 36].

Moreover, such systemic inflammatory status could also determine the high oxidative stress detected in PDA patients [35, 41]. In fact, according to some studies, reactive oxygen metabolites seem to be involved in ductal closure regulation and it has also been hypothesized that NSAID's activity in PDA closure could also be partly mediated by their ability in reactive species and oxidative stress reduction [28, 42–44].

In the study of Inayat et al. [45], in preterms with hsPDA, a poor antioxidant status within the first 48 hours after birth has been demonstrated through the detection of lower levels of superoxide dismutase (SOD), urinary catalase, and plasma and urinary 8-isoPGF2a, with an impairment in urinary prostaglandin E2, plasma and urinary thromboxane B2, and plasma SOD after pharmacological PDA treatment [45]. Further studies are needed to establish the correct relation between inflammation, oxidative stress, and PDA and to assess if there could be a possible role of such conditions related mediators in predicting PDA or monitoring therapeutic effects of closure treatment.

Metabolomic analysis also revealed a promising technique in the diagnosis and treatment of PDA; in fact, differently by the other mentioned mediators, it seems to have the singular ability to detect, at birth moment and only through the H-NMR evaluation of the first urine sample, the successive condition of persistent patency of DA at 3-4 days instead of its spontaneous closure and may also predict the exact individual response to the therapy. Moreover, different metabolic profiles of expression have been detected between responders and nonresponders to ibuprofen therapy. These interesting findings, though on small preterm groups, have been evidenced by preliminary results in the studies of Fanos et al. [46] and Castell Miñana et al. [47].

Further studies will help to fully understand the applicability fields of metabolomic holistic marker.

## 3. PDA Treatment

### 3.1. Indomethacin

Among nonselective COX inhibitors, intravenous (iv) indomethacin was the first drug used for PDA treatment, presenting a closure rate of about 70–85% without any other short-term benefits [26]. Since indomethacin has been used as a prophylaxis in PDA management, it has been shown to reduce the incidence of intraventricular hemorrhage (IVH ≥ grade 3 by 30%) and severe pulmonary hemorrhage by 35%, symptomatic PDA development, and necessity of surgical ligation [1, 14, 33, 48–52], without effects on mortality or long-term neurodevelopmental outcome [14].

Instead of this previous evidence, the recent prospective double cohort study of Liebowitz and Clyman [53], published on 2017, has pointed out also a protective effect of prophylactic indomethacin on development of BDP and death, instead of delayed PDA treatment (after 7 postnatal days) in extremely premature neonates.

However, for its high vasoconstrictor power, this drug has been associated with several side effects such as impairment in renal function until acute or chronic renal failure, oliguria, proteinuria, hyperkalemia [23], cerebral white matter damage, NEC, intestinal perforation (especially when coadministered with corticosteroids), and platelet dysfunction [2, 54].

Renal side effects are the most frequently reported and oliguria is generally reversible within 48 hours after last drug administration [23].

### 3.2. Ibuprofen

Recognizing these indomethacin related side effects, ibuprofen was subsequently introduced in the clinical practice, either orally or in iv manner; each course of therapy is composed of the standard dose of 10 mg/Kg/dose/day on the first day of treatment followed by two subsequent doses of 5 mg/Kg/dose/day on 2° and 3° days [33].

Ibuprofen shares with indomethacin the mechanism of action and the efficacy in PDA closure (success rate 70–85%) [26], but its lower vasoconstrictor effect leads to a reduced impact on microcirculation and consequent less impairment of renal function; this difference could be partly determined by a preferential effect of indomethacin on COX-1 instead of COX-2 but also by other mechanisms not exactly known [55, 56].

However, ibuprofen is not free from other significant side effects, such as pulmonary hypertension and hyperbilirubinemia [6, 9, 14].

Now it represents the first-choice drug for hsPDA treatment, but it is not recommended in prophylaxis because of the lack of efficacy in reducing intraventricular hemorrhage incidence, unlike indomethacin [1, 48, 49].

A recent randomized trial of Demir et al. [57], published on January 2017, has evaluated the ibuprofen intrarectal way of administration, which became as effective as the oral way in VLBW neonates with hsPDA. After treatment, in both groups the authors demonstrated higher levels of Cystatin-C, a biomarker of glomerular filtration which can suggest nephrotoxicity, indicating the necessity of a closely clinical observation especially in patients with a damaged renal function.

Higher doses of ibuprofen have been shown to improve closure rate, in particular using a treatment course of 20-10-10 mg/Kg/dose after standard doses failure, but the potential side effects of this drug regimen must be still clarified [14, 48]. Although some studies suggest the safety of high dose treatment [58], El-Mashad et al. [55] recommend the administration of low ibuprofen doses, underlying its inhibitory effect on hepatic glucuronidation of bilirubin and its high albumin binding affinity, which can increase the risk of bilirubin encephalopathy [59, 60].

Other studies show a major increase in Cystatin-C level after high dose regimen instead of standard doses [14, 48].

Drug responses can vary in different individuals, as it is known from pharmacogenomics. It is a current practice to administer the same ibuprofen dose for newborns of different GA and postnatal ages and this could result in a paradox from pharmacokinetic and pharmacodynamic perspective; however, other factors should be considered, such as the existence of two different ibuprofen enantiomers (S- and R-ibuprofen with half-lives of 25 and 10 hours, resp.) and the presence of genetic polymorphisms that can determine different clinical and side effects, dividing treated patients in extensive and poor metabolizers [9].

For example, polymorphisms in cytochromes P450 CYP2C8 and CYP2C9 are widespread in the population and some genetic variants can improve enzyme activity influencing NSAIDs metabolism, which represent their substrates. As reported by Agùndez et al. [61], individuals showing CYP2C8*∗*3 (rs11572080; rs10509681), CYP2C9*∗*2 (rs1799853), or CYP2C9*∗*3 (rs1057910) variations more frequently present gastrointestinal bleeding as an adverse effect of NSAIDs administration.

The possible role of CYP2C8 and 2C9 polymorphisms in influencing ibuprofen response, in preterm neonates treated for hsPDA, has been evaluated by Durrmeyer et al. [62]; in this study, similar drug responses have been reported among the studied individuals carrying different genetic variants, in a multivariate analysis; predictor factors for drug response seemed to be a higher gestational age and the non-Caucasian ethnicity, suggesting the possibility of this element influence on interindividual variability in ibuprofen response [62].

Moreover, other authors also evaluated P450 polymorphisms in healthy volunteers treated with ibuprofen; among these, Ochoa et al. [63] demonstrated the influence of CYP2C9*∗*2 and CYP2C9*∗*3 genetic variants on the pharmacokinetics of the enantiomers S-ibuprofen and R-ibuprofen and showed a possible gender predisposition in drug metabolism influence. In addition, Karaniewicz-Ada et al. [64] detected impaired ibuprofen enantiomers metabolism in individuals carrying CYP2C8*∗*3, CYP2C9*∗*2, and CYP2C9*∗*3 alleles, and also other studies evidenced the effect of CYP2C8*∗*3 variant on R-ibuprofen (with higher clearance) [65, 66] and fewer side effects in individuals showing CYP2C8*∗*3 variants [65].

More studies must be performed to fully understand the role of these purposed genetic determinants for ibuprofen responses. The goal of each pharmacotherapy would always be the administration of the exact individualized dose, showing the highest rate of success with the best safety profile and the lowest toxicity. In this perspective of person-based medicine, studies of pharmacokinetic and metabolomic are highly promising.

### 3.3. Nephrotoxicity of NSAIDs

Acute Kidney Injury (AKI) is an important issue in premature neonates of NICU, significantly contributing to morbidity and mortality of such critical population, since it is well known that these patients become more susceptible to kidney damage [67–71].

In preterms, AKI occurrence is generally a multifactorial event, but exposition to nephrotoxic medication plays an important role, potentially interfering with postnatal nephron generation and representing an avoidable cause of neonatal renal damage. Long-term effects of drug-induced AKI on both kidney function and general healthy outcome remain still understudied. However, recent data suggest that prematurity incomplete nephrogenesis, in addition to nephrotoxic administered toxins, could predispose to chronic kidney damage (CKD) [67, 72–74].

According to the recent study of Hanna et al. [67], published on 2016, the exact number of cases of nephrotoxin-associated AKI and its contribution on the development of CKD in neonatal population are not exactly known, for the lack of studies on a great number of patients but also for the absence of a systematic follow-up after neonatal AKI. Only small single center reports are actually available [75], but further studies will help to define the most appropriate application of nephrotoxic drugs and the correct surveillance in order to reduce the risk of CKD in treated patients [67].

The nephrotoxic effect of NSAIDs is related to prostaglandin important role during kidney and cardiovascular system adaptation after birth [23, 76, 77].

Prostaglandins neonatal circulating levels become higher than in successive life, since these mediators act as afferent arteriolar vasodilators and regulators of renal water clearance, facing the postnatal systemic resistances vasoconstriction. For these reasons, the inhibition of prostaglandin synthesis negatively affects renal blood flow and glomerular filtrate, generally resulting in transient oliguria [67, 78].

Moreover, neonatal kidney, not completely developed, is susceptible to the lack of prostaglandins. In fact, its maturation process closely depends on these mediators. This has been demonstrated both prenatally and in the postnatal period but appears more pronounced in preterms, whose urinary excretion of prostaglandin E2 (PGE2) and prostaglandin I2 (or prostacyclin) becomes higher than in neonates born with normal GA or with a month of postnatal life [23, 76, 77].

During studies conducted to evaluate NSAIDs renal damage, urinary PGE2 revealed a useful and noninvasive biomarker of nephrotoxicity, becoming significantly decreased in the urine of preterm infants after treatment for PDA closure, both with indomethacin and with ibuprofen, and this reduction became stronger in case of more severe side effects [23, 79].

Neonates with higher risk of nephrotoxic damage after ibuprofen administration are those with lower basal levels of PGE2; in the review of Fanos et al. [23] it is pointed out that no treatment should be considered in neonates with PGE2 urinary levels lower than 35 pg/ml; in neonates with rapid decrease of PGE2 urinary levels during treatment, ibuprofen suspension must be taken into account and neonates with PGE2 levels lower than 5 pg/ml after or during ibuprofen treatment will probably develop significant renal adverse effects [23].

Unlike this, the study of urinary isoprostanes, whose excretion increases in case of high oxidative status, revealed a lower level of these metabolites after ibuprofen administration, suggesting a possible protective effect of this NSAID against oxidative stress [23, 80].

Other sensitive and promising urinary biomarkers of kidney injury are represented by Cystatin-C and Neutrophil Gelatinase-Associated Lipocalin (NGAL); NGAL urinary excretion increases early during AKI and its detection becomes significantly helpful in monitoring nephrotoxicity in newborns [81].

### 3.4. Paracetamol

More recently, oral or iv administration of paracetamol (acetaminophen) gained attention in PDA treatment; the first case report on this topic has been published by Hammerman et al. on 2011 [82, 83]. Successively, this drug has been evaluated through many trials as safe and effective compared to traditional NSAIDs in PDA closure, with fewer side effects [2, 18, 84–86].

Before paracetamol introduction, in case of contraindication for NSAIDs, such as active or recent intracerebral hemorrhage (<48 h), thrombocytopenia (<50,000/mm^3^), bleeding diathesis (meaning INR > 1.5 and/or hematuria, blood in the stool, tracheal secretions or at the injection site), sepsis, NEC, intestinal perforation, pulmonary hemorrhage, hepatic damage with severe hyperbilirubinemia, renal dysfunction (oliguria <1 ml/kg/h also after adequate hydration, serum creatinine >110–140 *µ*mol, and BUN > 14 mmol/l), and hypersensitivity to ibuprofen [22, 48, 87], the only available solution was surgical ligation with all the connected risks [22, 55, 59]. 

However, further studies are needed before this drug can be recommended as first-line therapy; long-term outcomes of treatment and its possible late side effects at 18 or 24 months of postnatal age must be fully clarified [24, 26].

Yang et al. [88] demonstrated a probably higher renal safety of this drug describing a significantly lower reduction in PGE2 urinary excretion and minor incidence of oliguria comparing two groups of infants treated with paracetamol versus ibuprofen.

These advantages would be related to the different drug mechanism of action, because paracetamol is not a classical NSAID, having only a weak antiplatelet and anti-inflammatory activity. It exerts mainly central effects (analgesic, antipyretic) and reduces the synthesis of prostaglandins through the inhibition of prostaglandin synthetase (PGHS), as it happens with NSAIDs, but acting in a different enzyme site, called peroxidase region (POX) [12, 18, 55].

However, some hepatic side effects have been described after iv paracetamol administration, which may determine a transient increase in liver enzymes concentration [89] or, according to other studies, more serious acute liver toxicity events [48, 90–93].

Hepatotoxicity in neonates is not determined directly by paracetamol itself but can be caused by N-acetyl-p-benzoquinone imine (NAPQI) metabolite production by hepatic cytochrome P450 (CYP)-dependent mixed function oxidase enzyme. The mechanisms of NAPQI formation, sulphate elimination, and glucuronide production rate are still not exactly known in preterms [94, 95].

The hepatic paracetamol metabolism occurs through sulphation, glucuronidation, and oxidation. Administering therapeutic doses of paracetamol, glucuronidation, or sulphation is activated as first mechanism, producing nontoxic metabolites. Also hepatic oxidation of paracetamol by CYP1A2, 3A4, and 2E1 generates the highest reactive metabolite N-acetyl-p-benzoquinone imine (NAPQI) which is conjugated by glutathione into a renal metabolite that becomes safe. Instead, after an excessive dose of paracetamol, sulphation and glucuronidation pathways saturate and the resulting excessive dose of NAPQI consumes glutathione reserves becoming toxic [34]. It is well known that, in adults, the toxic paracetamol dose is about ten times higher than therapeutic concentration and paracetamol metabolism changes with the growth [96]; further evaluations could allow us to fully understand the extremely premature neonates metabolism [34].

It is described that neonates show an extremely variable glucuronidation rate and a limited ability for glutathione conjugation [97], with the predominance of sulphation [98], and that CYP is expressed early in postnatal life in full-term neonates while this is not well known in preterms [99].

However, clinical evidence shows a low or absent hepatic toxicity in neonates, suggesting the existence of a large therapeutic serum concentration range for paracetamol [34, 55, 100, 101].

This could depend on some mechanisms that seem to protect neonates in case of overdose such as slow oxidative metabolism and slow hepatic production of toxic metabolites and high rate of glutathione synthesis [48, 93, 102, 103].

N-acetylcysteine can detoxify NAPQI and becomes safe in neonates, so that it is used in case of subtoxic serum paracetamol concentration [94, 104] but there are no studies investigating its administration in PDA treatment [48].

For this lack of clear information about neonatal paracetamol metabolism, Cook et al. [95] performed a population pharmacokinetic model in order to define intravenous paracetamol effects and toxicity determinants and successively evaluated its predictive value with the aim of generalizing this knowledge to the whole neonates population. Their results evidenced that body weight (instead of gestational age, postmenstrual age, and unconjugated bilirubin levels) represents the principal predictor of intravenous paracetamol pharmacokinetics and the only covariate showing the adequate features to be included in the final proposed model, influencing both clearance and volume of drug distribution. According to these findings, the author suggests that the use of a parsimonious intravenous paracetamol dosage based on equivalent per kilogram (in all neonates, from extremely preterms to full-term newborns) could accommodate pharmacokinetics maturational changes, without the necessity to modify dosages and administration times according to gestational or postmenstrual age, as previously proposed by other studies. Cook et al. [95] also conclude with the observation that further studies will confirm if this simplified regimen really becomes unable to induce hepatotoxicity in all subcategories of neonates, considering the limited number of participants to the mentioned study but also the poor available knowledge about the real drug pharmacodynamics in neonates [95].

Serum paracetamol levels were evaluated in three studies of PDA management. In the study of Oncel et al. [105], these became 7.3 mcg/mL, 15.5 mcg/mL, and 14.7 mcg/mL during the three days of therapy. In the study of Yurttutan et al. [106], serum paracetamol levels after 24 h from administration became lower than 18 mcg/mL [48, 105–107].

Härkin et al. [108] analyzed 87 serum samples from 21 paracetamol treated patients and detected concentrations lower than 25.2 mg/L, without relevant accumulation. All these values resulted in therapeutic range for children (10–30 mcg/mL) [48, 107].

To examine the possible side effects of this drug, treated patients should be evaluated for alimentation disturbances, abdominal distension, oliguria, hypertension, and renal and hepatic functionality both during and after the treatment, also considering long-term consequences of clinical and subclinical side effects [94].

According to Tan and Baral [12], acetaminophen protein adducts or long chain acylcarnitines can be considered sensitive biomarkers helpful in monitoring the occurrence of potential hepatotoxic effects.

The effects of prophylactic paracetamol administration on PDA closure have been retrospectively evaluated by Aikio et al. [109] on 102 neonates born with <32 weeks of GA, demonstrating a reduction in PDA incidence from 30,7% to 14,7% after paracetamol introduction before the age of 72 hours of life, without an increase in adverse effects. However, more studies are needed to attest efficacy and safety of early PDA closure with paracetamol [109].

### 3.5. Surgical Ligation

Surgical closure of PDA, after failure of drug therapy or in case of contraindications to available drugs, is not exempt from adverse effects, such as vocal cord dysfunction, impaired neurological outcome, risk of BDP [14, 26, 110], retinopathy of premature (ROP), chylothorax and diaphragmatic paralysis, bleeding, pneumothorax, and cardiorespiratory failure [5].

For the high rate of complications related to PDA ligation, especially in terms of acute and severe hemodynamic side effects and worsening in neurodevelopmental outcomes, early prophylactic ligation is not recommended [33, 111, 112] and there is a spread trend of conservative ductal management, reserving this surgical approach only to those patients showing medical consequences of a large hsPDA after failure of two or more courses of medical treatments and those who need ventilator and oxygen support [14, 113–115].

Benitz and Committee on Fetus and Newborn [6] also underline that rapid and complete ductal closure occurring with ligation often leads to hemodynamic and respiratory complications, and supportive intensive care can be needed [6, 116].

## 4. Discussion and Recent Literature Review

Terrin et al. [82] performed on 2016 the first meta-analysis and systematic review on the results of the studies published between 2013 and 2014 evaluating paracetamol administration for PDA treatment (2 RCTs and 14 uncontrolled studies); the author reported a similar PDA closure rate of paracetamol instead of ibuprofen and a comparable safety profile, underlying that the analyzed studies included a relatively small number of neonates to consider these results as definitive [82].

In the same manner, the aim of this review is to discuss the recent published literature (2015-2016) evaluating paracetamol administration for hsPDA treatment in preterm neonates, comparing this drug to other NSAIDs or placebo or no intervention in order to add new evidence to what is already known about paracetamol efficacy and safety.

According to these features, we analyzed 15 studies (6 randomized controlled trials RCT, one of these is still ongoing, and 9 uncontrolled studies) (Table 1) and 16 reviews (Table 2), found between the articles in English language of MEDLINE using paracetamol, acetaminophen, Patent Ductus Arteriosus treatment, PDA, and preterm neonates as key words.

Data about the population characteristics (BW and GA, number of evaluated patients), type of study, kind and dose of administered drug, main outcome (closure rate), secondary outcomes (mortality, morbidity, or ductal reopening if mentioned), and safety profile for each mentioned study can be found in Tables 1 and 2.

On Table 3 we reported the different echocardiographic criteria to define hemodynamically significant PDA according to each author representing the cut-off for treatment; the table also shows the heterogeneous characteristics of the studied populations, which can make the results hardly comparable and must be taken in account during their interpretation. On Table 4, advantages, disadvantages, and side effects of PDA treatment strategies have been reported.

In the studies we discussed in this review, the standard dose of indomethacin (0,2 mg/Kg/dose/12 h for three doses), ibuprofen (10 mg/Kg/dose/day followed by 5 mg/Kg/dose/day on 2° and 3° days of therapy for 1–3 courses), or paracetamol (15 mg/Kg/dose/6 h for 3–7 days) has been used. Otherwise, we have indicated in the text the different dosage.

### 4.1. Results of Randomized Controlled Studies (2015-2016)

At first, we report the results of five randomized controlled trials (RCTs) performed administering paracetamol and attesting its efficacy in PDA closure, comparable to ibuprofen and indomethacin. Paracetamol has also showed a safer profile, with less side effects than NSAIDs [18, 55, 85, 88, 108].

A total of 641 preterms have been included and randomized; among these, 272 received paracetamol (149 oral versus 123 iv), 139 received iv indomethacin, 25 received placebo, 105 received oral ibuprofen, and 100 received iv ibuprofen.

Bagheri et al. [18] demonstrated comparable global closure rates between oral paracetamol and oral ibuprofen, with only minimal complications in paracetamol group. Considering its high safety, the author concluded paracetamol may be used as first-choice treatment but other studies should be performed to confirm it and to evaluate also spontaneous closure of PDA. In any case, we must underline the high GA (mean 31,53 weeks) of the newborns in this study, which could make these results hardly comparable with other trials.

In the study of Dash et al. [85], enteral paracetamol showed a PDA closure rate of 100% and no hepatotoxicity was detected. This surprising high result about paracetamol efficacy deviates from other studies' results, but it must be considered that this RCT evaluated patients showing a mean GA of 31,6 weeks, higher than neonates in other trials.

In contrast to many authors' results, Dash et al. [85] reported a high intestinal bleeding occurrence in paracetamol group (26.3%) and this result, according to El-Mashad et al. [55], could be influenced by the high osmolality of paracetamol used in their trial. The authors confirmed the global safety of enteral paracetamol treatment and concluded attesting its validity in preterms hsPDA management; however they affirm that more data are needed, especially long-term studies, to evaluate neurodevelopmental outcome effects of paracetamol administration [85].

Härkin et al. [108] demonstrated a faster hsPDA closure rate in paracetamol group (95%) than in placebo group. The authors used a different drug dosage, administering 20 mg/kg of paracetamol at 24 hours of life, followed by 7,5 mg/kg every 6 h for 4 days and the ductus closed at a mean of 177 hours of postnatal life in treated patients versus 338 hours in controls. However, GA influenced ductal closure; in fact, in extremely preterm infants (<27 weeks' GA), paracetamol did not show a significant effect; among these, 4 preterms (50%) required PDA ligation. Paracetamol seemed to be more efficacious in males than in females; this result did not show a statistical relevance but, among the studies considered in this review, this is the only trial evaluating and pointing out a difference in gender drug response. No adverse effects or signs of hepatotoxicity have been described and serum paracetamol concentration became as a result included in the safety range, showing that early iv paracetamol accelerates hsPDA closure without relevant side effects.

Yang et al. [88] demonstrated a similar PDA closure rate between oral paracetamol and ibuprofen, but less adverse events were detected in newborns receiving paracetamol, with lower incidence of oliguria. In conclusion this study evidenced lower toxicity, also corresponding to lower plasma and urinary PGE2 levels, in paracetamol group.

El-Mashad et al. [55] performed the first large prospective randomized study comparing the efficacy and side effects of paracetamol, ibuprofen, and indomethacin simultaneously. For this purpose, *n* = 300 neonates have been enrolled and treated with iv paracetamol (*n* = 100), iv ibuprofen (*n* = 100), or iv indometacin (*n* = 100). Global PDA closure rate did not show significant differences among the three groups and an improvement in ventilatory setting was also demonstrated after successful PDA closure. In NSAIDs groups the authors detected a significant increase in creatinine and serum blood urea nitrogen levels associated with a significant platelet count and urine output (UOP) reduction. Only among the ibuprofen treated patients was there also a significant increase in bilirubin levels. The effect in platelet reduction is absent after paracetamol treatment and this could be explained, according to the authors, by its lack of action on thromboxane, unlike NSAIDs. In conclusion, in this study, paracetamol has shown the same efficacy of indomethacin and ibuprofen in preterm neonates PDA closure but less side effects, especially for its low impact on renal function, platelet count, and GI bleeding.

### 4.2. Results of Uncontrolled Studies (2015-2016)

Below, we report major results and findings of analyzed uncontrolled studies. A total of *n* = 202 patients have been treated with paracetamol and, among these, *n* = 2 showed hepatotoxicity.

The efficacy of paracetamol after ibuprofen failure has been evaluated in two trials by Roofthooft et al. [22] and Valerio et al. [87], finding different results. According to Roofthooft et al. [22], paracetamol is not effective in hsPDA closure in newborns with a postnatal age > 2 weeks, showing a global paracetamol success of 18% in the studied sample, significantly lower than literature data. However different effectiveness levels have been detected in various categories of treated patients: closure rate (or PDA diameter reduction) was 46% after paracetamol first-choice therapy, while this drug failed when administered after two cycles of ibuprofen. In this second group of patients, paracetamol showed 100% of failure, suggesting that, after ineffective ibuprofen treatment, paracetamol in VLBW is not effective in closing PDA, maybe for its late administration (mean 14 days) or for the patients features in this study (lower GA than other trials or variability in the hemodynamic significance criteria among the studies, showed in Table 3), although it became a safe treatment, without renal or hepatic side effects.

In the study of Valerio et al. [87], comparing the efficacy of paracetamol between a “rescue” group (after failure of ibuprofen) and a “first-line” group (contraindication for ibuprofen), no significant difference in PDA closure efficacy was detected. More patients of the “first-line” group underwent PDA surgical ligation (26.7%) and showed PDA reopening (10 versus 0%); it could also depend on more critical conditions of these patients. GA represented a closure predictor in “first-line” group and, in attempt to obtain a predictor index for PDA closure, Valerio and his collaborators demonstrated the role of CRIB score (a clinical score resulting in combination of GA, BW, sex, patient's temperature, and maximum base excess in the first 12 h of life) [130], as an independent predictor of PDA closure in “rescue” group. The authors conclude confirming the efficacy of iv paracetamol for PDA closure in VLBW and ELBW preterm population and they also suggest the oral route seems to be valid but not recommended for such infants, showing intestinal mucosa immaturity with consequent unpredictable absorption.

Instead of the results of Roofthooft and Valerio and his group [87] attested paracetamol efficacy also when administered after ibuprofen failure, suggesting the necessity of other trials to improve our knowledge.

Paracetamol efficacy becomes greater when started in the first week of life and this may be related to the higher prostaglandin circulating levels during early postnatal life; moreover, efficacy of paracetamol when administered after NSAIDs failure could be also depend on an additive effect [82].

Four small case series have been performed by Tekgündüz et al. [122], Memisoglu et al. [118], Peňa-Juárez et al. [123], and Tofé Valera et al. [117] on 13, 11, 10, and 3 preterms, finding a hsPDA closure rate of 76.9%, 90,9%, 70%, and 100%, respectively, after paracetamol administration. Globally, hepatotoxicity (hypertransaminasemia) was described in two patients and no other significant side effects were detected.

The retrospective cohort studies of Weisz et al. [120] and Mohanty et al. [121], including, respectively, 26 and 40 patients, also demonstrated a positive response in paracetamol treated preterms for hsPDA in 46% and 72,5% of neonates in absence of complications, demonstrating that paracetamol could be a safe therapy in such infants.

The two different administration routes of paracetamol have been compared by Sancak et al. [119] in 18 VLBW newborns; hsPDA closure rate seemed to be higher in those treated with oral compared to iv paracetamol administration after two courses of therapy, but this result was not statistically significant. Both the treatments did not show hepatic toxicity. In the future, larger trials should be performed in order to define the possible differences between the two administration routes.

### 4.3. Results of Reviews (2015-2016)

Our analysis on 16 reviews investigating the role of paracetamol in hsPDA treatment (Table 2) shows that most authors support the efficacy of this drug in ductal closure, becoming comparable to NSAIDs, with inconstant transient lower side effects (in terms of elevation of liver enzymes) instead of GI bleeding, oliguria, and hyperbilirubinemia showed after ibuprofen therapy [12, 14, 24, 34, 48, 82, 118, 127, 129]. However, an appropriated monitoring in order to early detect paracetamol toxicity is recommended [34].

In the review of Tan and Baral [12], among *n* = 88 treated patients, *n* = 6 showed transitory liver enzymes elevation [12].

A safer profile in terms of gastrointestinal bleeding and hyperbilirubinemia after paracetamol administration instead of ibuprofen has been described by Evans [14] and Terrin et al. [82].

All these authors agree with the possibility of using paracetamol in case of ibuprofen or indomethacin contraindications and/or failure but other studies are needed to confirm the safety profile of this therapy, to establish the lowest effective dose and to evaluate long-term outcomes, in particular the possibility of neurocognitive impairment, before considering it as the drug of first choice [12, 24, 34, 127].

Le et al. [129] agree with the idea that paracetamol seems to be a good alternative in PDA treatment and should be considered, in case of ibuprofen contraindication, before ligation. The author also recommends performing other trials because two studies published on 2013 found low iv paracetamol success rate [89, 131] in small groups of patients (*n* = 29 and *n* = 3); moreover, the high mean postnatal age (22 days) at paracetamol administration in the study of Roofthooft et al. must be also considered [131].

According to the reviews of Oncel et al. [48] and Terrin et al. [82], paracetamol efficacy would be lower in extremely preterm neonates (<28 weeks of GA), probably for structural limitations in these subjects, which present a higher expression of prostaglandin receptors in the wall of the ductus and a thin-walled DA, with a lower represented neointimal mounds. In these patients, administration of PG inhibitors can be followed by functional closure but less frequently by the structural ductal closure [48].

El Kuffash et al. [31] evaluated late treatment with iv paracetamol beyond the 2nd week of life which became effective in hsPDA closure, avoiding PDA ligation. Anyway, more studies are needed to confirm the role of paracetamol late administration.

Evans [14] described a similar reopening rate after PDA treatment both in paracetamol and in ibuprofen groups and also concluded underlying the same efficacy of the two drugs, which should be confirmed through other trials.

### 4.4. Results about Conservative Treatment

Results in support of conservative PDA treatment have been reported by Slaughter et al. [132] in their cohort study published on January 2017 and by Letshwiti et al. [133]. The first is a large study on *n* = 12.018 newborns (≤28 weeks' GA) affected by PDA, evaluated and treated in 25 different hospitals. The 32% of all infants have been treated with NSAIDs (27% received indomethacin and 7% ibuprofen) and no association was demonstrated between NSAID treatment (performed between 2 and 28 postnatal days) and the odds of mortality or BPD, mortality, or moderate-severe BPD at 36 weeks of postmenstrual age versus similar not treated preterms. The lack of definition of echocardiographic PDA features in treated and not treated population could represent a limit that makes this study about the conservative approach difficult to compare to other studies' evidence.

The study of Letshwiti et al. [133], published on December 2016, compares three different PDA management approaches: early targeted treatment, symptomatic treatment, and no treatment. Infants in symptomatic treatment group were treated in case of hsPDA. Patients in early targeted group received ibuprofen in case large PDA is evaluated by echocardiography in the first 48 h; in conservative treatment group PDA was managed with increased PEEP and fluid restriction. The author demonstrated a lower rate of ibuprofen therapy and ligation in conservative group and a significantly decreased incidence of chronic lung disease compared to symptomatic treatment group (18% versus 51%) and to patients early treated (18% versus 46%).

### 4.5. Results Evaluating Neurocognitive Impact

Most authors affirm the necessity to assess the long-term neurocognitive impact of PDA treatment, because it could represent a limitation in PDA management: a reduction in this outcome could support a conservative approach to PDA. In this regard, Janz-Robinson et al. [134] reported data of a retrospective population-based cohort study attesting the association between PDA treatment (medical or surgical) and risk of developmental delay, cerebral palsy, sensorineural or conductive deafness, or bilateral blindness instead of nontreated patients, at age 2-3 years, especially for neonates <25 weeks' GA. These results may validate a permissive tolerance of PDA.

On the contrary, the results of the follow-up study of Oncel et al. [135], published on 2017, attest there are no differences in neurodevelopmental outcomes at 18 and 24 months in *n* = 61 ex preterm infants (GA < or = 30 weeks), treated for PDA with oral ibuprofen or paracetamol [135].

These results suggest the necessity to fully understand the exact relation between treatment and neurodevelopmental outcome before recommending the most appropriate strategy for PDA management.

## 5. Patent Ductus Arteriosus and Cardiovascular Programming

Several recent studies pointed out the association between risk of adult life cardiovascular diseases and insults occurring during fetal growth and in very early postnatal period; the concept of “developmental programming” has been proposed to explain this correlation [136, 137].

According to this theory, each condition of fetal adaptation to an inadequate or deprived environment during embryonal development can constitute an insult that will make the individual more susceptible and vulnerable throughout the whole life, as a permanent adverse effect [136].

This happens because fetal and early postnatal life are “critical periods” in which tissues development is realized through high cells proliferation rate, and each occurring adverse event can conduce to negative consequences in organs growth [136, 138]. All these correlations are best demonstrated on animals models, but there is also much evidence in epidemiological human studies [136].

It is known that adverse events in the first phases of life could influence blood pressure and determinate cardiovascular disease in adult life [136, 139–142]; moreover, low birth weight may be associated with long-term cardiovascular diseases [143–146].

It is in this perspective that we consider the correct management of PDA fundamental, a cardiovascular pathology occurring in the critical prematurity period, whose incorrect treatment could potentially lead to negative “programming” with permanent cardiovascular function impairment and a variable impact on heart health.

## 6. Conclusions and Future Perspectives

The goal of the studies on PDA management would be to perform an individualized therapy, choosing the sartorial treatment for each of the patient characteristics, which could be the most effective as much as possible, personalized, and with the lowest side effects.

Between the available drugs for PDA treatment, paracetamol seems to be a promising alternative and most authors agree with the necessity of more trials to establish the safer dose in preterms and its efficacy. If these arguments will be confirmed, paracetamol could become the first-line therapy for hsPDA treatment.

However, non-well designed trials about proper paracetamol dosing or large randomized controlled trials (RCT) on short and long-term safety have been published in the last two years; as a consequence, we are still waiting for these important data before thinking to change the actual standard care for PDA into paracetamol.

In the next future we hope that “omics” technologies, namely, metabolomics, could be useful to assess and fully clarify the different metabolic pathways of mediators able to early predict, in an individualized way, closure of PDA allowing the avoidance of drug toxicity or a profile of metabolites able to predict the exact response to treatment or drug safety, as it is known that the high interindividual variation in therapy response is strongly related to the subject's biochemical state.

## Figures and Tables

**Table 1:** Experimental studies investigating paracetamol administration in PDA treatment (2015-2016).

Authors	Year	Patients	GA (weeks)	BW (gr)	Treatment	Study	Dose	Results	Side effects
Härkin et al. [108]	2016	48	<32	Mean: 1.220 Para group, 1.120 placebo group	iv Para (*n* = 23) versus placebo	Controlled trial, fase I.II, double blind, randomized	20 mg/kg at 24 h followed by 7,5 mg/kg every 6 h for 4 days	Faster closure in Para group (95% CI 0.25–0.97, *P* = 0.016). Mean (95% CI) postnatal closure age 177 h (31.1–324) for Para versus 338 h (118–557) for controls (*P* = 0.045) GA > 27 w: mean postnatal closure age 80 h in Para group versus h placebo 322 h (*P* = 0.004). GA < 27 w: *n* = 8, not Para effect on ductus (*P* = 0.63), 4 (50%) required PDA ligation (Para *n* = 3, placebo *n* = 1). Para closure in males (HR 0.31, 95% CI 0.12–0.85, *P* = 0.023), higher than females (95% CI 0.27–1.96, *P* = 0.52)	No adverse effects or hepatotoxicity

Valerio et al. [87]	2016	48	23–32	Mean: “first-line” group 853,3 ± 286,9; “rescue” group 887,7 ± 297	iv Para (*n* = 48) (*n* = 30 for ibu contraindications. *N* = 18 after ibu failure)	Observational longitudinal prospective study	ibu 10-5-5 mg/Kg versus Para 15 mg/kg every 6 h	No significant differences in closure rate “first-line” versus “rescue” groups after 2 (56.7% versus 61.1%, *P* = 0.7624) or 3 cycles (63.3 versus 77.8% *P* = 0.2959).^(1)^ Surgical ligation rate higher in “first-line” (26.7%) versus “rescue” group (16.7%). Reopening rate In “first-line” higher versus “rescue” group (10 versus 0%), and 91% of needing surgical closure had GA ≤27 w	No hepatotoxicity

Bagheri et al. [18]	2016	129	<37	Mean: 1.646,26 Para group, 1.642,62 ibu group	Oral Para (*n* = 67) versus oral ibu (*n* = 62)	Randomized trial	ibu 20-10-10 mg/Kg versus Para 15 mg/kg every 6 h	After 1° course of treatment: closure in 82.1% *n* = 55 patients oral Para vs. 75.8% *n* = 47 oral Ibu (*P* = 0.38). 2° course: closure oral Para group versus oral ibu group 50% versus 73.3% (*P* = 0.21)	No significant complications for both drugs

Dani et al. [84]	2016	110	25–31+^6^	—	Para iv (*n* = 55) versus ibu iv (*n* = 55)	Randomized multicenter controlled study	Para 15 mg/kg/dose every 6 h for 3 days. Ibu 10-5-5 mg/kg/day	On course	—

Tofé Valera et al. [117]	2016	3	<32	<1.900	3 iv Para for ibu contraindications	Case series	Para 15 mg/kg every 6 h for 3–6 days	100% closure (3/3 Patients)	Hypertransaminasemia *n* = 1 patient

Yang et al. [88]	2016	87	<37	Mean: Para group 2.219 ± 606, ibu group 2.091 ± 657	Oral ibu (*n* = 43) versus oral Para (*n* = 44)	Randomized controlled trial	10-5-5 mg/kg ibuprofen versus Para 15 mg/kg every 6 h for 3 days	Plasma and urinary PGE2 levels in Para group (45.0 ± 36.9 ng/l) significantly lower than ibu group (73.5 ± 44.8 ng/l, *P* = 0.002). Closure rate similar between Para (*n* = 31, 70.5%) and ibu groups (=33, 76.7%, *P* = 0.50). Lower oliguria in Para group (*n* = 1, 2.3%) than ibu group (*n* = 6, 14.0%)	Low adverse events in Para group

Memisoglu et al. [118]	2016	11	23–30+^3^	415–1.580	iv Para for contraindications to Ibu or Indo	Case series	15 mg/kg every 6 h for 3 days	Closure rate 90.9% (*n* = 10/11)	No adverse or side effects

Sancak et al. [119]	2016	18	n.k.	<1.500	iv Para (*n* = 10) versus oral Para (*n* = 8)	Retrospective study	15 mg/kg every 6 h for 3 days	After 2 courses of treatment, higher closure rate in oral Para group versus iv Para group (88% versus 70%), not statistically significant (*P* = 0.588)	Liver function tests normal

Weisz et al. [120]	2016	26	<28	n.k.	Oral Para for Indo failure	Retrospective cohort study	15 mg/kg every 6 h for 3–7 days	Echo indices improved in 46% (*n* = 12) infants (3 closed and 9 reduced to mild shunt), all avoiding ligation. No echo improvement in 54% (*n* = 14) infants (*n* = 8/14 underwent ligation)	No differences in 2^ry^ outcomes

Mohanty et al. [121]	2016	40	<32	n.k.	Para for ibu contraindication	Prospective study	n.k.	72.5% (*n* = 29/40) successful closure versus 29.5% (*n* = 11/40) not response	No major complications

El-Mashad et al. [55]	2016	300	<28	<1.500	iv Para (*n* = 100) versus iv ibu (*n* = 100) or iv Indo (*n* = 100)	Prospective randomized Study	Para 15 mg/kg/6 h iv. Ibu iv 10 mg/kg on 1° day, 5 mg/kg on the 2° and 3° days. 0.2 mg/kg/12 h ind iv for 3 doses	No significant difference in closure rate (*P* = 0.868). Cumulative closure rate higher after 2° course (88% in Para group, 83% in ibu group, 87% in Indo group). After closure, improvement in ventilatory settings (*P* < 0.001)	Serum creatinine levels and BUN higher in Indo versus ibu group (*P* value < 0.001). PLT and UOP lower in Indo than ibu group (*P* < 0.001). Increase in bilirubin levels in ibu group (*P* < 0,05 ). No difference of HB level or liver enzymes (*P* > 0.05). GIT bleeding rate higher in Indo and Ibu groups (*P* < 0.05)

Roofthooft et al. [22]	2015	33	<28	<1.500	Para for contraindication to ibu or failure of treatment	Prospective observational single center Study	ibu 1 or 2 courses (10 mg/kg on 1° day, 5 mg/kg on the 2° and 3° days). Para iv (15 mg/kg/6 h) 3–7 days	Group A: 46% Para efficacy Groups B-C: Para treatment ineffective in all patients (2 died, in the others surgical ligation)^(2)^. After previous exposure to ibu, Para ineffective in 100%	No adverse effects

Dash et al. [85]	2015	77	Mean: 28,5 Para group, 28,9 ibu group	≤1.500	Enteral Para (*n* = 38) versus iv Indo (*n* = 39)	Randomized controlled trial	Para per os (15 mg/kg/dose, 6 hourly for 7 days) or iv Indo (0.2 mg/kg/dose, once daily for 3 days)	Closure rate 100% (*n* = 36/36) in enteral Para group versus 94.6% (*n* = 35/37) in iv Indo group (*P* = 0.13). 2° outcomes similar in the two groups. 26,3% of GI bleeding in Para group	No hepatotoxicity in Para group

Tekgündüz et al. [122]	2015	13	24–31	470–1.390	iv Para for contraindication to oral ibu	Case series	*N* = 1 patient 15 mg/kg/dose every 6 h *N* = 12 patients, 10 mg/kg/dose every 6 h for 3 or 4 days	PDA closure in 76.9% (*n* = 10/13)	Hepatotoxicity *N* = 1 patient

Peňa-Juárez et al. [123]	2015	10	30–36	840–1.600	Oral Para	Case series	15 mg/kg/dose every 6 h for 1-2 days	Closure in *n* = 7/10 patients (70%); *n* = 3/10 (30%) patients underwent surgical ligation *N* = 2 patients died (causes not related to drug administration)	No significant changes in liver function and platelet count

Para = paracetamol. Ibu = ibuprofen. Indo= indomethacin. iv = intravenous. GA = gestational age. BW= birth weight. w = weeks. gr = grams. BUN = serum blood urea nitrogen. UOP = urine output. HB = hemoglobin. PGE2 = Prostaglandin E2. GI = gastrointestinal. n.k. = not known data. (1) “First-line” group: paracetamol as first-choice therapy for ibuprofen contraindications. “Rescue” group: paracetamol after ibuprofen failure or for development of contraindications during its administration. (2) Group A: paracetamol first choice for ibuprofen primary contraindications. Group B: paracetamol after ibuprofen incomplete courses for development of contraindications during its administration. Group C: paracetamol after failure of two complete ibuprofen courses.

**Table 2:** Reviews investigating paracetamol administration in PDA treatment (2015-2016).

Authors	Year	Patients	GA (weeks)	BW (gr)	Treatment	Study	Dose	Results	Side effects
Benitz and Committee on Fetus and Newborn [6]	2016	n.e.	n.e.	n.e.	n.e.	n.e.	n.e.	Absent long term outcomes improvement for early routine PDA closure in preterms	Rapid, complete therapeutic closure often leads to severe hemodynamic and respiratory collapse

Oncel and Erdeve [48]	2016	149	<30	n.e.	Oral or iv Para for Ibu contraindication or failure (after 2 courses)	7 Studies	Para 15 mg/Kg every 6 hours for 3 days	Closure rates similar Para versus Ibu. GA < 28 w: reduced efficacy of Para	Less GI bleeding/jaundice in Para group. Transient increase in liver enzymes after iv Para. No differences in other side effects

Tan and Baral [12]	2016	88	23–37	n.e.	Oral or iv Para	12 Studies	Para 15 mg/Kg every 6 hours for 3 days	Closure rate 76.1% (*n* = 67/88)	Transitory liver enzymes elevation in 6 patients

Vettukattil [33]	2016	n.e.	n.e.	n.e.	n.e.	n.e.	Para 15 mg/Kg every 6 hours for 3 days. Ibu 10-5-5 mg/Kg on 1° day, 5 mg/kg on the 2° and 3° days	Evidence supporting intervention in PDA closure in extremely preterms within 72 h of birth	n.e.

Rostas and McPherson [34]	2016	n.e.	n.e.	n.e.	n.e.	n.e.	Para 15 mg/kg/dose every 6 hours for 3–6 days	Para reasonable strategy for PDA treatment if COX inhibitors contraindicated or failure. The same efficacy enteral Para versus enteral Ibu	Increase GI bleeding in Ibu group. Appropriate monitoring for toxicity is required during Para administration

van den Anker and Allegaert [24]	2016	n.e.	n.e.	n.e.	n.e.	n.e.	n.e.	No indication for prophylactic therapy of PDA	Less adverse effects of Para versus Ibu or Indo

Sivanandan and Agarwal [124]	2016	n.e.	n.e.	n.e.	Para indicated in infants with NSAIDs contraindications	n.e.	n.e.	Similar efficacy Para versus NSAIDs (limited data available from randomized trials). Actually, Para not first choice, more trials needed	n.e.

Bancalari and Jain [125]	2016	n.e.	n.e.	n.e.	n.e.	n.e.	n.e.	Prophylactic Indo reduce vasopressor-dependent hypotension in preterms. More studies needed to evaluate transient hypotension long-term consequences on respiratory or neurologic outcomes	n.e.

Chandrasekaran [126]	2016	n.e.	n.e.	n.e.	n.e.	n.e.	n.e.	Para seems effective and safer than COX inhibitors in closing PDA	n.e.

Terrin et al. [82]	2016	371	n.e.	n.e.	Para for Ibu resistance or contraindication	Meta-analysis on 14 uncontrolled studies and 2 RCTs	30–60 mg/kg/die	GA < 28 w: reduced efficacy of Para (67% closure rate). GA ≥ 28 w: 89% closure rate; RCTs showed no significant difference Para versus Ibu groups for mortality, morbidity or ductal reopening	Para: safer profile GI bleeding (95% CI = 0.07–1.03), hyperbilirubinemia (95% CI = 0.34–0.97), oliguria (95% CI = 0.25–1.79.) Para: transient increase in ASTs or *γ*GT in a small n° of patients

Ohlsson and Shah [127]	2015	250	n.e.	n.e.	Oral Para versus oral Ibu	Meta-analysis on 2 large RCTs	n.e.	No significant difference oral Para versus oral Ibu in closure rate after 1° course (95% CI 0.67, 1.22). No significant differences Para versus Ibu groups in 2^ry^ outcomes except duration for hyperbilirubinemia and need of supplemental oxygen	Lower in Para group

Evans [14]	2015	250	<36 (*n* = 90 < 30 w)	n.e.	Oral Para	2 Studies	Para 15 mg/kg/dose. Ibu 10-5-5 mg/Kg on 1° day, 5 mg/kg on the 2° and 3° days	Similar efficacy Para versus Ibu in 2 RCT (79% versus 81% and 77% versus 72%). Reopening rate 1° RCT (24.1% versus 16.1%; *P* = 0.43 Para versus Ibu group). Para group (*n* = 1 patient) Ibu group (*n* = 2) surgical ligation (GA 26 w ). 2° RCT closure rate: Para 56.3% (*n* = 45), Ibu 47.5% (*n* = 38 *P* = 0.268). Reopening rate *n* = 5 and *n* = 6 of Para versus Ibu. *N* = 4 in each group closure after 2° course	Less collateral GI effects (bleeding/jaundice) in Para group

Jain and Shah [128]	2015	n.e.	n.e.	n.e.	Oral Para versus oral Ibu	2 RCTs	Para 15 mg/kg per dose every 6 hours for 2 to 7 days	No differences Para versus Ibu closure rate or 2^ry^ outcomes: (1° study, 95% CI, 0.60–1.15 68 and 2° study, 95% CI 0.57–2.62). NSAIDs first choice therapy but Para safe option	No difference in side effects

El-Khuffash et al. [31]	2015	36	24^+4^–27^+6^	645–954	Para iv	Retrospective	15 mg/kg/die for 3–6 days	Para closure rate 25% (*n* = 9/31). No responders 11% (*n* = 4/31). PDA ligation 64% (*n* = 23/31). Median days PDA constriction 0.9 days [0.5–1.9] post-treatment. PDA definitely closed at discharge *n* = 30/31. *N* = 10 infants received Ibu prior to Para treatment. In this cohort, Ibu not efficacious in any infant. *N* = 4/9 infants pretreated with Ibu:PDA closure (44%)	No side effects

Le et al. [129]	2015	338	n.e.	n.e.	Ibu resistance or contraindication	12 studies, 2 RCTs	15 mg/kg/dose every 6 h for 3 days	>76% success PDA closure (case reports); PDA closure rates from 72.5 to 81.2% in RCTs	Few incidents of elevated liver enzymes

n.e. = not explained in the review. Para = paracetamol. Ibu = ibuprofen. Indo = indomethacin. iv = intravenous. GA = gestational age. BW = birth weight. w = weeks. gr = grams. GI = gastrointestinal. NSAIDs = nonsteroidal anti-inflammatory drugs. COX = cyclooxygenase.

**Table 3:** Echocardiographic criteria to define hemodynamically significant PDA (hsPDA), representing the cut-off for treatment according to the authors, and Heterogeneous characteristics of the studied populations.

Authors	Year	PDA size	LA/Ao ratio	(i) Studied population (ii) Postnatal age at diagnosis	(i) Birth weight (ii) Gestational age
Roofthooft et al.^1^ [22]	2015	>2 mm	>1,6	(i) 33 VLBW with these echo features (ii) Median 51 days group A, 30 days group B, none in group C^2^	(i) <1500 g (ii) <28 w

Dash et al. [85]	2015	≥1,5 mm	>1,5	(i) 77 preterms with these echo features (ii) <48 hours	(i) ≤1500 (ii) Mean: 28,5 w Para group, 28,9 w Ibu group

Peňa-Juárez et al.^3^ [123]	2015	≥1 mm	>1,8	(i) 10 preterms with these echo features (ii) <10 days	(i) 840–1600 g (ii) 30–36 w

Härkin et al. [108]	2016	Diameter > 50% LPA	>1,4	(i) Among 63 screened VLBW patients, 48 had these echo features and underwent randomization (76,2%) (ii) n.e.	(i) Mean: 1220 g Para group, 1120 placebo group (ii) <32 w

Valerio et al.^4^ [87]	2016	≥1,4 mm/Kg	≥1,4	(i) Among the 196 studied preterms, 102 had PDA (52%), and, among these patients, 48 (47,1%) had these echo features (ii) Echo performed at 48–72 hours	(i) Mean: 853,3 g “first-line” group, 887,7 g “rescue group”^5^ (ii) 23–32 w

Bagheri et al. [18]	2016	>1,5 mm	>1,2	(i) 160 patients enrolled for hsPDA but 31 excluded. Final group: 129 patients (ii) ≤14 days	(i) Mean: 1646 g Para group, 1642 g Ibu group (ii) <37 w

El-Mashad et al. [55]	2015	>1,5 mm	>1,6	(i) 300 preterms with these echo features (ii) ≤ 14 days	<1500 g <28 w

Dani et al. [84]	2016	>1,5 mm	>1,3	(i) On course (ii) 48–72 hours	(i) n.e. (ii) 25–31^+6^ w

Tofé Valera et al. [117]	2016	>2 mm	>1,5	(i) 3 preterms (ii) 3, 5 and 14 days	(i) <1900 g (ii) <32 w

Yang et al. [88]	2016	>1,4 mm	>1,4	(i) Among 96 neonates with these echo features, 87 underwent randomization (ii) 15 hours–10 days	(i) Mean: 2091 g Ibu group, 2219 g Para group (ii) <37 w

Memisoglu et al. [118]	2016	>1.4 mm/kg	>1.4	(i) 11 preterms (ii) n.k.	(i) 415–1580 g (ii) 23–30^+3^ w

Benitz and Committee on Fetus and Newborn [6]	2016	≥1,5 mm	≥1,5	(i) n.e. (ii) n.e.	(i) n.e. (ii) n.e.

Tan and Baral [12]	2016	≥1,4 mm	≥1,5	(i) n.e. (ii) n.e.	(i) n.e. (ii) 25–37 w

Vettukattil [33]	2016	>1.4 mm	>1.4	(i) n.e. (ii) n.e.	(i) n.e. (ii) n.e.

PDAsize: mm or mm/Kg; PA = Pulmonary Artery; La/Ao ratio = left atrium/aorta diameter; LVO/FVC = Left ventricular output and systemic flow through superior vena cava; LPA = Left Pulmonary Artery; Qp/Qs = Pulmonary/Systemic Flow Ratio; Para = paracetamol; Ibu = ibuprofen; echo = echocardiographic; w = weeks; g = grams; n.e. = not explained in the text; n.k. = not known data; ^1^PDA/LPA > 0,8; ^2^Group A: paracetamol first choice for ibuprofen primary contraindication. Group B: paracetamol after ibuprofen incomplete courses for development of contraindication. Group C: paracetamol after failure of two complete ibuprofen courses; ^3^Qp/Qs ratio > 1,8; ^4^LVO/FVC ratio ≥ 4. ^5^“First-line” group: paracetamol as first-choice therapy for ibuprofen contraindications. “Rescue” group: paracetamol after ibuprofen failure or for development of contraindications during its administration.

**Table 4:** Comparison among different strategies of PDA treatment.

	No treatment	Indomethacin	Ibuprofen	Paracetamol	Surgical ligation
Advantages	(i) Avoid drug exposition (ii) PDA could close spontaneously	(i) In PDA prophylaxis, it reduces intraventricular hemorrhage (IVH) incidence (30%) and early pulmonary hemorrhage (35%), development of symptomatic PDA, necessity of surgical ligation (ii) Efficacy in PDA closure	(i) Efficacy in PDA closure (ii) Lower nephrotoxicity than indomethacin	(i) Efficacy in PDA closure (ii) Lower side effects instead of NSAIDs	(i) Rapid and complete ductal closure

Disadvantages	Possible lack of closure	(i) Toxicity (ii) Contraindications: active or recent hemorrhage, thrombocytopenia, sepsis, NEC, intestinal perforation, hepatic damage with severe hyperbilirubinemia, renal dysfunction	(i) Toxicity (ii) Contraindications: active or recent hemorrhage, thrombocytopenia, sepsis, NEC, intestinal perforation, hepatic damage with severe hyperbilirubinemia, renal dysfunction	(i) Toxicity still to be fully defined (ii) Long term outcomes still to be fully defined	(i) Risks of an invasive procedure (ii) Long term outcomes still to be fully defined

Standardization of dosages	—	0,2 mg/Kg/dose/12 h	10 mg/Kg/dose/day followed by 5 mg/Kg/dose/day on 2° and 3° days of therapy	15 mg/Kg/dose/6 h	—

Standardization of therapy length	—	3 doses	1–3 courses	3–7 days	—

Route of administration	—	Intravenous	(i) Intravenous (ii) oral	(i) Intravenous (ii) oral	—

Need of monitoring	—	Yes, especially for nephrotoxicity	Yes, especially for nephro- and hepatotoxicity	Yes, especially for hepatotoxicity	Yes, rapid and complete ductal closure can lead to hemodynamic and respiratory complications

Side effects	Respiratory distress syndrome (RDS), prolonged need for ventilation, pulmonary hemorrhage, bronchopulmonary dysplasia (BDP), necrotizing enterocolitis (NEC), renal function damage, intraventricular hemorrhage (IVH), periventricular leukomalacia (PLV), cerebral palsy, death	Nephrotoxicity (until acute or chronic renal failure), cerebral white matter damage, necrotizing enterocolitis (NEC), intestinal perforation, platelet dysfunction	Nephrotoxicity, pulmonary hypertension, hyperbilirubinemia, necrotizing enterocolitis (NEC), intestinal perforation, platelet dysfunction	Transient and inconstant increase in liver enzymes	Hemodynamic side effects, cardiorespiratory failure, risk of BDP, retinopathy of prematurity (ROP), vocal cord dysfunction, chylothorax, diaphragmatic paralysis, bleeding, pneumothorax, impaired neurological outcome

## Additional Points

*What Is Known*. (i) In preterm infants, spontaneous closure of Ductus Arteriosus (DA) often fails or is delayed, and this condition can be associated with several short- and long-term complications. (ii) Nonsteroidal anti-inflammatory drugs (NSAIDs), traditionally used in Patent Ductus Arteriosus (PDA) treatment, are related to several side effects especially in terms of nephrotoxicity.

*What Is New*. (i) The results of the studies analyzed in this review mostly support paracetamol efficacy in ductal closure, showing inconstant low and transient elevation in liver enzymes as side effect. (ii) Other studies are needed to confirm the safety profile of this therapy, to establish the lowest effective dose, and to evaluate long-term outcomes before considering it the drug of first choice.

## References

[1] Fanos V., Pusceddu M., Dessì A., Marcialis M. A. (2011). Should we definitively abandon prophylaxis for patent ductus arteriosus in preterm new-borns?. *Clinics*.

[2] Mitra S., Florez I. D., Tamayo M. E. (2016). Effectiveness and safety of treatments used for the management of patent ductus arteriosus (PDA) in preterm infants: A protocol for a systematic review and network meta-analysis. *BMJ Open*.

[3] Gittenberger-de Groot A. C. (1977). Persistent ductus arteriosus: most probably a primary congenital malformation. *British Heart Journal*.

[4] Chuaqui B. J., Piwonka G. C., FarrÚ O. A. (1977). The wall in persistent ductus arteriosus. *Virchows Archiv A Pathological Anatomy and Histology*.

[5] Abdel-Hady H., Nasef N., Shabaan A. E., Nour I. (2013). Patent ductus arteriosus in preterm infants: Do we have the right answers?. *BioMed Research International*.

[6] Benitz W. E., Committee on Fetus and Newborn (2016). Patent Ductus Arteriosus in Preterm Infants. *Pediatrics*.

[7] Bhattacharya M., Asselin P., Hardy P. (1999). Developmental changes in prostaglandin E2 receptor subtypes in porcine ductus arteriosus: Possible contribution in altered responsiveness to prostaglandin E2. *Circulation*.

[8] Bouayad A., Kajino H., Waleh N. (2001). Characterization of PGE2 receptors in fetal and newborn lamb ductus arteriosus. *American Journal of Physiology - Heart and Circulatory Physiology*.

[9] Antonucci R., Bassareo P., Zaffanello M., Pusceddu M., Fanos V. (2010). Patent ductus arteriosus in the preterm infant: new insights into pathogenesis and clinical management. *Journal of Maternal-Fetal and Neonatal Medicine*.

[10] Clyman R. I., Mauray F., Roman C., Heymann M. A., Payne B. (1983). Factors determining the loss of ductus arteriosus responsiveness to prostaglandin E. *Circulation*.

[11] Abrams S. E., Walsh K. P., Coker S. J., Clarkson M. J. (1995). Responses of the post-term arterial duct to oxygen, prostaglandin E 2, and the nitric oxide donor, 3-morpholinosydnonimine, in lambs and their clinical implications. *Heart*.

[12] Tan Z. H., Baral V. R. (2016). Principles of clinical management of patent ductus arteriosus in extremely preterm neonates. *Current Pediatric Reviews*.

[13] Waleh N., Kajino H., Marrache A. M. (2004). Prostaglandin E2-mediated relaxation of the ductus arteriosus: Effects of gestational age on G protein-coupled receptor expression, signaling, and vasomotor control. *Circulation*.

[14] Evans N. (2015). Preterm patent ductus arteriosus: a continuing conundrum for the neonatologist?. *Seminars in Fetal and Neonatal Medicine*.

[15] Koch J., Hensley G., Roy L., Brown S., Ramaciotti C., Rosenfeld C. R. (2006). Prevalence of spontaneous closure of the ductus arteriosus in neonates at a birth weight of 1000 grams or less. *Pediatrics*.

[16] Jhaveri N., Moon-Grady A., Clyman R. I. (2010). Early surgical ligation versus a conservative approach for management of patent ductus arteriosus that fails to close after indomethacin treatment. *The Journal of pediatrics*.

[17] Schena F., Francescato G., Cappelleri A. (2015). Association between hemodynamically significant patent ductus arteriosus and bronchopulmonary dysplasia. *Journal of Pediatrics*.

[18] Bagheri M. M., Niknafs P., Sabsevari F. (2016). Comparison of oral acetaminophen versus ibuprofen in premature infants with patent ductus arteriosus. *Iranian Journal of Pediatrics*.

[19] Benitz W. E. (2010). Treatment of persistent patent ductus arteriosus in preterm infants: Time to accept the null hypothesis. *Journal of Perinatology*.

[20] Lipman B., Server G. A., Brazy J. E. (1982). Abnormal cerebral haemodynamics in preterm infants with patent ductus arteriosus. *Pediatrics*.

[21] Investigators of the Vermont-Oxford Trials Network Database Project (1993). The Vermont- Oxford Trials Network: very low birth weight outcomes for 1990. *Pediatrics*.

[22] Roofthooft D. W. E., van Beynum I. M., de Klerk J. C. A. (2015). Limited effects of intravenous paracetamol on patent ductus arteriosus in very low birth weight infants with contraindications for ibuprofen or after ibuprofen failure. *European Journal of Pediatrics*.

[23] Fanos V., Marcialis M. A., Bassareo P. (2011). Renal safety of non steroidal anti inflammatory drugs (NSAIDs) in the pharmacologic treatment of patent ductus arteriosus. *Journal of Maternal-Fetal and Neonatal Medicine*.

[24] van den Anker J. N., Allegaert K. (2016). Acetaminophen to Prevent Symptomatic Patent Ductus Arteriosus: Another Drug Bites the Dust?. *Journal of Pediatrics*.

[25] Demirel G., Erdeve O., Dilmen U. (2012). Pharmacological management of PDA: Oral versus intravenous medications. *Current Clinical Pharmacology*.

[26] Allegaert K., Anderson B., Simons S., Van Overmeire B. (2013). Paracetamol to induce ductus arteriosus closure: Is it valid?. *Archives of Disease in Childhood*.

[27] Hammerman C., Bin-Nun A., Kaplan M. (2012). Managing the Patent Ductus Arteriosus in the Premature Neonate: A New Look at What We Thought We Knew. *Seminars in Perinatology*.

[28] Van Overmeire B., Chemtob S. (2005). The pharmacologic closure of the patent ductus arteriosus. *Seminars in Fetal and Neonatal Medicine*.

[29] Clyman R. I., Couto J., Murphy G. M. (2012). Patent Ductus Arteriosus: Are Current Neonatal Treatment Options Better or Worse Than No Treatment at All?. *Seminars in Perinatology*.

[30] Bose C. L., Laughon M. M. (2007). Patent ductus arteriosus: Lack of evidence for common treatments. *Archives of Disease in Childhood: Fetal and Neonatal Edition*.

[31] El-Khuffash A. F., Slevin M., McNamara P. J., Molloy E. J. (2011). Troponin T, N-terminal pro natriuretic peptide and a patent ductus arteriosus scoring system predict death before discharge or neurodevelopmental outcome at 2 years in preterm infants. *Archives of Disease in Childhood: Fetal and Neonatal Edition*.

[32] Czernik C., Metze B., Müller C., Bührer C. (2013). Urinary NT-proBNP and ductal closure in preterm infants. *Journal of Perinatology*.

[33] Vettukattil J. J. (2016). Patent ductus arteriosus in extremely premature neonates. *Current Pediatric Reviews*.

[34] Rostas S. E., McPherson C. C. (2016). Pharmacotherapy for patent ductus arteriosus: Current options and outstanding questions. *Current Pediatric Reviews*.

[35] Hillman M., Meinarde L., Rizzotti A., Cuestas E. (2015). Inflammation, high sensitività C-Reactive protein and persistent patent ductus arteriosus in preterm infants. *Revista Espanola de Cardiologia (English Edition)*.

[36] Kim E. S., Kim E.-K., Choi C. W. (2010). Intrauterine inflammation as a risk factor for persistent ductus arteriosus patency after cyclooxygenase inhibition in extremely low birth weight infants. *Journal of Pediatrics*.

[37] Rodrigo F. G.-M., Henríquez G. G., Aloy J. F., Pérez A. G.-A. (2014). Outcomes of very-low-birth-weight infants exposed to maternal clinical chorioamnionitis: A multicentre study. *Neonatology*.

[38] Gonzalez A., Sosenko I. R. S., Chandar J., Hummler H., Claure N., Bancalari E. (1996). Influence of infection on patent ductus arteriosus and chronic lung disease in premature infants weighing 1000 grams or less. *Journal of Pediatrics*.

[39] Irving K., David S., Marina M. (2010). A unifying biologic explanation for "high-sensitivity" C-reactive protein and "low-grade" inflammation. *Arthritis Care and Research*.

[40] Meinarde L., Hillman M., Rizzotti A., Basquiera A. L., Tabares A., Cuestas E. (2016). C-reactive protein, platelets, and patent ductus arteriosus. *Platelets*.

[41] Chang B. A., Huang Q., Quan J. (2011). Early inflammation in the absence of overt infection in preterm neonates exposed to intensive care. *Cytokine*.

[42] Akar M., Yildirim T. G., Sandal G. (2016). Does ibuprofen treatment in patent ductus arteriosus alter oxygen free radicals in premature infants?. *Cardiology in the Young*.

[43] Clyman R. I., Saugstad O. D., Mauray F. (1989). Reactive oxygen metabolites relax the lamb ductus arteriosus by stimulating prostaglandin production. *Circulation Research*.

[44] Hlller K.-O., Wilson R. L. (1983). Hydroxyl-free radicals and anti-inflammatory drugs: biological inactivation studies and reaction rate constants. *Biochemical Pharmacology*.

[45] Inayat M., Bany-Mohammed F., Valencia A. (2015). Antioxidants and Biomarkers of Oxidative Stress in Preterm Infants with Symptomatic Patent Ductus Arteriosus. *American Journal of Perinatology*.

[46] Fanos V., Barberini L., Antonucci R., Atzori L. (2012). Pharma-metabolomics in Neonatology: Is it a Dream or a Fact?. *Current Pharmaceutical Design*.

[47] Castell Miñana M., Quintás Soriano G., Fernández Tudela B., Vento Torres M. Urinary metabolomics as a new strategy to discriminate response to ibuprofen therapy in preterm neonates with patent ductus arteriosus.

[48] Oncel M. Y., Erdeve O. (2016). Oral medications regarding their safety and efficacy in the management of patent ductus arteriosus. *World Journal of Clinical Pediatrics*.

[49] Ment L. R., Oh W., Ehrenkranz R. A., Philip A. G. (1994). Low-dose indomethacin and prevention of intraventricular hemorrhage: a multicenter randomized trial. *Pediatrics*.

[50] Rolland A., Shankar-Aguilera S., Diomandé D., Zupan-Simunek V., Boileau P. (2015). Natural evolution of patent ductus arteriosus in the extremely preterm infant. *Archives of Disease in Childhood: Fetal and Neonatal Edition*.

[51] Fowlie P. W., Davis P. G., McGuire W. (2010). Prophylactic intravenous indomethacin for preventing mortality and morbidity in preterm infants. *Cochrane database of systematic reviews (Online)*.

[52] Alfaleh K., Smyth J. A., Roberts R. S., Solimano A., Asztalos E. V., Schmidt B. (2008). Prevention and 18-month outcomes of serious pulmonary hemorrhage in extremely low birth weight infants: Results from the trial of indomethacin prophylaxis in preterms. *Pediatrics*.

[53] Liebowitz M., Clyman R. I. (2017). Profilactic Indomethacin compared with delayed conservative management of the patent ductus arteriosus in extremely preterm infants: effects on neonatal outcomes. *The Journal of Pediatrics*.

[54] Irmesi R., Marcialis M. A., Anker J. V. D., Fanos V. (2014). Non-steroidal anti-inflammatory drugs (NSAIDs) in the management of patent ductus arteriosus (PDA) in preterm infants and variations in attitude in clinical practice: A flight around the world. *Current Medicinal Chemistry*.

[55] El-Mashad A. E.-R., El-Mahdy H., El Amrousy D., Elgendy M. (2016). Comparative study of the efficacy and safety of paracetamol, ibuprofen, and indomethacin in closure of patent ductus arteriosus in preterm neonates. *European Journal of Pediatrics*.

[56] Vane J. R., Botting R. M. (1997). Mechanism of action of anti-inflammatory drugs. *Advances in Experimental Medicine and Biology. Plenum Press*.

[57] Demir N., Peker E., Ece I., Balahoroğlu R., Tuncer O. (2017). Efficacy and safety of rectal ibuprofen for patent ductus arteriosus closure in very low birth weight preterm infants. *The Journal of Maternal-Fetal & Neonatal Medicine*.

[58] Meißner U., Chakrabarty R., Topf H.-G., Rascher W., Schroth M. (2012). Improved closure of patent ductus arteriosus with high doses of ibuprofen. *Pediatric Cardiology*.

[59] Rheinlaender C., Helfenstein D., Walch E., Berns M., Obladen M., Koehne P. (2009). Total serum bilirubin levels during cyclooxygenase inhibitor treatment for patent ductus arteriosus in preterm infants. *Acta Paediatrica, International Journal of Paediatrics*.

[60] Zecca E., Romagnoli C., De Carolis M. P., Costa S., Marra R., De Luca D. (2009). Does ibuprofen increase neonatal hyperbilirubinemia?. *Pediatrics*.

[61] Agùndez J. A. G., Garcia-Martin E., Martinez C. (2009). Genetically based impairment in CYP2C8- and CYP2C9-dependent NSAID metabolism as a risk factor for gastrointestinal bleeding: Is a combination of pharmacogenomics and metabolomics required to improve personalized medicine?. *Expert Opinion on Drug Metabolism and Toxicology*.

[62] Durrmeyer X., Hovhannisyan S., Médard Y. (2010). Are cytochrome P450 CYP2C8 and CYP2C9 polymorphisms associated with ibuprofen response in very preterm infants?. *PLoS ONE*.

[63] Ochoa D., Prieto-Pérez R., Román M. (2015). Effect of gender and CYP2C9 and CYP2C8 polymorphisms on the pharmacokinetics of ibuprofen enantiomers. *Pharmacogenomics*.

[64] Karaniewicz-Ada M., Uczak M., Głwka F. (2009). Pharmacokinetic studies of enantiomers of ibuprofen and its chiral metabolites in humans with different variants of genes coding CYP2C8 and CYP2C9 isoenzymes. *Xenobiotica*.

[65] López-Rodríguez R., Novalbos J., Gallego-Sandín S. (2008). Influence of CYP2C8 and CYP2C9 polymorphisms on pharmacokinetic and pharmacodynamic parameters of racemic and enantiomeric forms of ibuprofen in healthy volunteers. *Pharmacological Research*.

[66] Martinez C., Garcia-Martin E., Blanco G. (2005). The effect of the cytocrome P450 CYP2C8 polymorphism on the disposition of (R)-ibuprofen enantiomer in healty subjects. *British Journal of Clinical Pharmacology*.

[67] Hanna M. H., Askenazi D. J., Selewski D. T. (2016). Drug-induced acute kidney injury in neonates. *Current Opinion in Pediatrics*.

[68] Askenazi D. J., Griffin R., McGwin G., Carlo W., Ambalavanan N. (2009). Acute kidney injury is independently associated with mortality in very low birthweight infants: a matched case: control analysis. *Pediatric Nephrology*.

[69] Gadepalli S. K., Selewski D. T., Drongowski R. A., Mychaliska G. B. (2011). Acute kidney injury in congenital diaphragmatic hernia requiring extracorporeal life support: An insidious problem. *Journal of Pediatric Surgery*.

[70] Carmody J. B., Swanson J. R., Rhone E. T., Charlton J. R. (2014). Recognition and reporting of aki in very low birth weight infants. *Clinical Journal of the American Society of Nephrology*.

[71] Zwiers A. J. M., de Wildt S. N., Hop W. C. J. (2013). Acute kidney injury is a frequent complication in critically ill neonates receiving extracorporeal membrane oxygenation: A 14-year cohort study. *Critical Care*.

[72] Askenazi D. J., Feig D. I., Graham N. M., Hui-Stickle S., Goldstein S. L. (2006). 3–5 year longitudinal follow-up of pediatric patients after acute renal failure. *Kidney International*.

[73] Mammen C., Al Abbas A., Skippen P. (2012). Long-term risk of CKD in children surviving episodes of acute kidney injury in the intensive care unit: a prospective cohort study. *The American Journal of Kidney Diseases*.

[74] Zaffanello M., Bassareo P. P., Cataldi L., Antonucci R., Biban P., Fanos V. (2010). Long-term effects of neonatal drugs on the kidney. *Journal of Maternal-Fetal and Neonatal Medicine*.

[75] Abitbol C. L., Bauer C. R., Montané B., Chandar J., Duara S., Zilleruelo G. (2003). Long-term follow-up of extremely low birth weight infants with neonatal renal failure. *Pediatric Nephrology*.

[76] Antonucci R., Cuzzolin L., Arceri A., Fanos V. (2007). Urinary prostaglandin E2 in the newborn and infant. *Prostaglandins and Other Lipid Mediators*.

[77] Agostiniani R., Mariotti P., Cataldi L. (2002). Role of renal PGE2 in the adaptation from foetal to extrauterine life in term and preterm infants. *Prostaglandins Leukotrienes and Essential Fatty Acids*.

[78] Antonucci R., Zaffanello M., Puxeddu E. (2012). Use of non-steroidal anti-inflammatory drugs in pregnancy: impact on the fetus and newborn. *Current Drug Metabolism*.

[79] Antonucci R., Cuzzolin L., Arceri A., Dessì A., Fanos V. (2009). Changes in urinary PGE2 after ibuprofen treatment in preterm infants with patent ductus arteriosus. *European Journal of Clinical Pharmacology*.

[80] Longini M., Perrone S., Vezzosi P. (2011). Isoprostane levels in urine of preterm newborns treated with ibuprofen for patent ductus arteriosus closure. *Pediatric Nephrology*.

[81] Fanos V., Antonucci R., Zaffanello M., Mussap M. (2012). Nenatal drug induced nephrotoxicity: Old and next generation biomarkers for early detection and management of neonatal drug-induced nephrotoxicity, with special emphasis on uNGAL and on metabolomics. *Current Medicinal Chemistry*.

[82] Terrin G., Conte F., Oncel M. Y. (2016). Paracetamol for the treatment of patent ductus arteriosus in preterm neonates: A systematic review and meta-analysis. *Archives of Disease in Childhood: Fetal and Neonatal Edition*.

[83] Hammerman C., Bin-Nun A., Markovitch E., Schimmel M. S., Kaplan M., Fink D. (2011). Ductal closure with paracetamol: A surprising new approach to patent ductus arteriosus treatment. *Pediatrics*.

[84] Dani C., Poggi C., Mosca F. (2016). Efficacy and safety of intravenous paracetamol in comparison to ibuprofen for the treatment of patent ductus arteriosus in preterm infants: Study protocol for a randomized control trial. *Trials*.

[85] Dash S. K., Kabra N. S., Avasthi B. S., Sharma S. R., Padhi P., Ahmed J. (2015). Enteral paracetamol or intravenous indomethacin for closure of patent ductus arteriosus in preterm neonates: A randomized controlled trial. *Indian Pediatrics*.

[86] Dang D., Wang D., Zhang C., Zhou W., Zhou Q., Wu H. (2013). Comparison of oral paracetamol versus ibuprofen in premature infants with patent ductus arteriosus: A randomized controlled trial. *PLoS ONE*.

[87] Valerio E., Valente M. R., Salvadori S., Frigo A. C., Baraldi E., Lago P. (2016). Intravenous paracetamol for PDA closure in the preterm: a single-center experience. *European Journal of Pediatrics*.

[88] Yang B., Gao X., Ren Y., Wang Y., Zhang Q. (2016). Oral paracetamol vs. oral ibuprofen in the treatment of symptomatic patent ductus arteriosus in premature infants: A randomized controlled trial. *Experimental and Therapeutic Medicine*.

[89] Alan S., Kahvecioglu D., Erdeve O., Atasay B., Arsan S. (2013). Is Paracetamol a useful treatment for ibuprofen-resistant patent ductus arteriosus?. *Neonatology*.

[90] Dart R. C., Rumack B. H. (2012). Intravenous acetaminophen in the United States: Iatrogenic dosing errors. *Pediatrics*.

[91] Nevin D. G., Shung J. (2010). Intravenous paracetamol overdose in a preterm infant during anesthesia. *Paediatric Anaesthesia*.

[92] Isbister G. K., Bucens I. K., Whyte I. M. (2001). Paracetamol overdose in a preterm neonate. *Archives of Disease in Childhood - Fetal and Neonatal Edition*.

[93] Porta R., Sánchez L., Nicolás M., García C., Martínez M. (2012). Lack of toxicity after paracetamol overdose in a extremely preterm neonate. *European Journal of Clinical Pharmacology*.

[94] Oncel M. Y., Erdeve O. (2015). Safety of therapeutics used in management of patent ductus arteriosus in preterm infants. *Current Drug Safety*.

[95] Cook S. F., Roberts J. K., Samiee-Zafarghandy S. (2016). Population Pharmacokinetics of Intravenous Paracetamol (Acetaminophen) in Preterm and Term Neonates: Model Development and External Evaluation. *Clinical Pharmacokinetics*.

[96] Allegaert K., Anderson B. J., Naulaers G. (2004). Intravenous paracetamol (propacetamol) pharmacokinetics in term and preterm neonates. *European Journal of Clinical Pharmacology*.

[97] Allegaert K., de Hoon J., Verbesselt R., Vanhole C., Devlieger H., Tibboel D. (2005). Intra- and interindividual variability of glucuronidation of paracetamol during repeated administration of propacetamol in neonates. *Acta Paediatrica*.

[98] Manyike P. T., Kharasch E. D., Kalhorn T. F., Slattery J. T. (2000). Contribution of CYP2E1 and CYP3A to acetaminophen reactive metabolite formation. *Clinical Pharmacology and Therapeutics*.

[99] Kearns G. L., Abdel-Rahman S. M., Alander S. W., Blowey D. L., Leeder J. S., Kauffman R. E. (2003). Developmental pharmacology - Drug disposition, action, and therapy in infants and children. *New England Journal of Medicine*.

[100] Allegaert K., Rayyan M., De Rijdt T., Van Beek F., Naulaers G. (2008). Hepatic tolerance of repeated intravenous paracetamol administration in neonates. *Paediatric Anaesthesia*.

[101] Jacqz-Aigrain E., Anderson B. J. (2006). Pain control: Non-steroidal anti-inflammatory agents. *Seminars in Fetal and Neonatal Medicine*.

[102] Anderson B. J., Allegaert K. (2009). Intravenous neonatal paracetamol dosing: The magic of 10 days. *Paediatric Anaesthesia*.

[103] Palmer G. M., Atkins M., Anderson B. J. (2008). I.V. acetaminophen pharmacokinetics in neonates after multiple doses. *British Journal of Anaesthesia*.

[104] Beringer R. M., Thompson J. P., Parry S., Stoddart P. A. (2011). Intravenous paracetamol overdose: Two case reports and a change to national treatment guidelines. *Archives of Disease in Childhood*.

[105] Oncel M. Y., Yurttutan S., Degirmencioglu H. (2013). Intravenous paracetamol treatment in the management of patent ductus arteriosus in extremely low birth weight infants. *Neonatology*.

[106] Yurttutan S., Oncel M. Y., Arayici S. (2013). A different first-choice drug in the medical management of patent ductus arteriosus: Oral paracetamol. *Journal of Maternal-Fetal and Neonatal Medicine*.

[107] Kratz A., Ferraro M., Sluss P. M., Lewandrowski K. B. (2004). Case records of the massachusetts general hospital. weekly clinicopathological exercises. laboratory reference values. *The New England Journal of Medicine*.

[108] Härkin P., Härmä A., Aikio O. (2016). Paracetamol Accelerates Closure of the Ductus Arteriosus after Premature Birth: A Randomized Trial. *Journal of Pediatrics*.

[109] Aikio O., Härkin P., Saarela T., Hallman M. (2014). Early paracetamol treatment associated with lowered risk of persistent ductus arteriosus in very preterm infants. *Journal of Maternal-Fetal and Neonatal Medicine*.

[110] Sung S. I., Chang Y. S., Chun J. Y. (2016). Mandatory Closure Versus Nonintervention for Patent Ductus Arteriosus in Very Preterm Infants. *Journal of Pediatrics*.

[111] Nemerofsky S. L., Parravicini E., Bateman D., Kleinman C., Polin R. A., Lorenz J. M. (2008). The ductus arteriosus rarely requires treatment in infants > 1000 grams. *American Journal of Perinatology*.

[112] Wickremasinghe A. C., Rogers E. E., Piecuch R. E. (2012). Neurodevelopmental outcomes following two different treatment approaches (early ligation and selective ligation) for patent ductus arteriosus. *Journal of Pediatrics*.

[113] McNamara P. J., Stewart L., Shivananda S. P., Stephens D., Sehgal A. (2010). Patent ductus arteriosus ligation is associated with impaired left ventricular systolic performance in premature infants weighing less than 1000 g. *Journal of Thoracic and Cardiovascular Surgery*.

[114] Noori S., Friedlich P., Seri I., Wong P. (2007). Changes in Myocardial Function and Hemodynamics after Ligation of the Ductus Arteriosus in Preterm Infants. *Journal of Pediatrics*.

[115] Kabra N. S., Schmidt B., Roberts R. S., Doyle L. W., Papile L., Fanaroff A. (2007). Neurosensory Impairment after Surgical Closure of Patent Ductus Arteriosus in Extremely Low Birth Weight Infants: Results from the Trial of Indomethacin Prophylaxis in Preterms. *Journal of Pediatrics*.

[116] Teixeira L. S., Shivananda S. P., Stephens D., Van Arsdell G., McNamara P. J. (2008). Postoperative cardiorespiratory instability following ligation of the preterm ductus arteriosus is related to early need for intervention. *Journal of Perinatology*.

[117] Tofé Valera I., Jaraba Caballero M. P., Ruiz Gonzalez M. D., Rodrıguez Benıtez M. V., Parraga Quiles M. J. (2016). he role of paracetamol for closing Patent Ductus Arteriosus. A challenging alternative for ductal closure?. *Revista Espanola de Cardiologia (English Edition)*.

[118] Memisoglu A., Alp Ünkar Z., Cetiner N. (2016). Ductal closure with intravenous paracetamol: A new approach to patent ductus arteriosus treatment. *Journal of Maternal-Fetal and Neonatal Medicine*.

[119] Sancak S., Yildirim T. G., Topcuoglu S., Yavuz T., Karatekin G., Ovali F. (2016). Oral versus intravenous paracetamol: Which is better in closure of patent ductus arteriosus in very low birth weight infants?. *Journal of Maternal-Fetal and Neonatal Medicine*.

[120] Weisz D. E., Martins F. F., Nield L. E., El-Khuffash A., Jain A., McNamara P. J. (2016). Acetaminophen to avoid surgical ligation in extremely low gestational age neonates with persistent hemodynamically significant patent ductus arteriosus. *Journal of Perinatology*.

[121] Mohanty P. K., Nagesh N. K., Razak A. (2016). Oral paracetamol for closure of patent ductus arteriosus in selected preterm neonates. *Indian Pediatrics*.

[122] Tekgündüz K. Ş., Ceviz N., Caner I. (2015). Intravenous paracetamol with a lower dose is also effective for the treatment of patent ductus arteriosus in pre-term infants. *Cardiology in the Young*.

[123] Peňa-Juárez R. A., de Medina-Andra M. A., MartÍnez-González M. T., Gallardo-Meza A. F., Cortez-Comparan D., Piňa Garay M. A. (2015). Ductus Arteriosus closure with paracetamol: a pilot study. *Revista Espanola de Cardiologia (English Edition)*.

[124] Sivanandan S., Agarwal R. (2016). Pharmacological Closure of Patent Ductus Arteriosus: Selecting the Agent and Route of Administration. *Pediatric Drugs*.

[125] Bancalari E., Jain D. (2017). Management of Patent Ductus Arteriosus: Are We Looking at the Right Outcomes?. *The Journal of Pediatrics*.

[126] Chandrasekaran A. (2016). A Survey among Neonatologists on the Management of Patent Ductus Arteriosus. *Indian Journal of Pediatrics*.

[127] Ohlsson A., Shah P. S. (2015). Paracetamol (acetaminophen) for patent ductus arteriosus in pretermor low-birth-weight infants. *The Cochrane database of systematic reviews*.

[128] Jain A., Shah P. S. (2015). Diagnosis, evaluation, and management of patent ductus arteriosus in preterm neonates. *JAMA Pediatrics*.

[129] Le J., Gales M. A., Gales B. J. (2015). Acetaminophen for Patent Ductus Arteriosus. *Annals of Pharmacotherapy*.

[130] Ezz- Eldin Z. M., Abdel Hamid T. A., Labib Youssef M. R., Nabil H. E.-D. (2015). Clinical Risk Index for Babies (CRIB II) Scoring System in Prediction of Mortality in Premature Babies. *Journal of Clinical and Diagnostic Research*.

[131] Roofthooft D. W. E., Van Beynum I. M., Helbing W. A., Reiss I. K. M., Simons S. H. P. (2013). Paracetamol for ductus arteriosus closure: Not always a success story. *Neonatology*.

[132] Slaughter J. L., Reagan P. B., Newman T. B., Klebanoff M. A. (2017). Comparative effectiveness of nonsteroidal anti-inflammatory drug treatment vs no treatment for Patent Ductus Arteriosus in preterm infants. *JAMA Pediatrics*.

[133] Letshwiti J. B., Semberova J., Pichova K., Dempsey E. M., Franklin O. M., Miletin J. (2016). A conservative treatment of patent ductus arteriosus in very low birth weight infants. *Early Human Development*.

[134] Janz-Robinson E. M., Badawi N., Walker K., Bajuk B., Abdel-Latif M. E. (2015). Neurodevelopmental outcomes of premature infants treated for Patent Ductus Arteriosus: A population-based cohort study. Neonatal intensive care units network. *The Journal of Pediatrics*.

[135] Oncel M. E., Eras Z., Uras N., Canpolat F. E., Erdeve O., Oguz S. (2017). Neurodevelopmental outcomes of preterm infants treated with oral paracetamol versus ibuprofen for patent ductus arteriosus. *American Journal of Perinatology*.

[136] Zohdi V., Lim K., Pearson J. T., Jane Black M. (2015). Developmental programming of cardiovascular disease following intrauterine growth restriction: Findings utilising a rat model of maternal protein restriction. *Nutrients*.

[137] Hales C. N., Barker D. J. P. (2001). The thrifty phenotype hypothesis. *British Medical Bulletin*.

[138] Lucas A., Fewtrell M. S., Cole T. J. (1999). Fetal origins of adult disease—the hypothesis revisited. *British Medical Journal*.

[139] Barker D. J., Osmond C., Golding J., Kuh D., Wadsworth M. E. (1989). Growth in utero, blood pressure in childhood and adult life, and mortality from cardiovascular disease. *British Medical Journal*.

[140] Barker D. J. P., Bull A. R., Osmond C., Simmonds S. J. (1990). Fetal and placental size and risk of hypertension in adult life. *British Medical Journal*.

[141] Barker D. J., Martyn C. N. (1997). The fetal origins of adult hypertension. *Advances in Nephrology from the Necker Hospital*.

[142] Eriksson J. G., Forsén T. J., Kajantie E., Osmond C., Barker D. J. P. (2007). Childhood growth and hypertension in later life. *Hypertension*.

[143] Taylor S. J., Whincup P. H., Cook D. G., Papacosta O., Walker M. (1997). Size at birth and blood pressure: Cross sectional study in 8–11 year old children. *British Medical Journal*.

[144] Yiu V., Buka S., Zurakowski D., McCormick M., Brenner B., Jabs K. (1999). Relationship between birthweight and blood pressure in childhood. *American Journal of Kidney Diseases*.

[145] Blake K. V., Gurrin L. C., Evans S. F. (2000). Maternal cigarette smoking during pregnancy, low birth weight and subsequent blood pressure in early childhood. *Early Human Development*.

[146] Adair L., Dahly D. (2005). Developmental determinants of blood pressure in adults. *Annual Review of Nutrition*.

